# Diagnostics in Ebola Virus Disease in Resource-Rich and Resource-Limited Settings

**DOI:** 10.1371/journal.pntd.0004948

**Published:** 2016-10-27

**Authors:** Robert J Shorten, Colin S Brown, Michael Jacobs, Simon Rattenbury, Andrew J. Simpson, Stephen Mepham

**Affiliations:** 1 Public Health Laboratory Manchester, Manchester Royal Infirmary, Manchester, United Kingdom; 2 University College London, Centre for Clinical Microbiology, Department of Infection, London United Kingdom; 3 Hospital for Tropical Diseases, University College London Hospital, London, United Kingdom; 4 King’s Sierra Leone Partnership, King’s Centre for Global Health, King’s College London, and King’s Health Partners, London, United Kingdom; 5 Department of Infection, Royal Free London NHS Foundation Trust, London, United Kingdom; 6 Rare and Imported Pathogens Laboratory, Public Health England, Salisbury, United Kingdom; University of Geneva Hospitals, SWITZERLAND

## Abstract

The Ebola virus disease (EVD) outbreak in West Africa was unprecedented in scale and location. Limited access to both diagnostic and supportive pathology assays in both resource-rich and resource-limited settings had a detrimental effect on the identification and isolation of cases as well as individual patient management. Limited access to such assays in resource-rich settings resulted in delays in differentiating EVD from other illnesses in returning travellers, in turn utilising valuable resources until a diagnosis could be made. This had a much greater impact in West Africa, where it contributed to the initial failure to contain the outbreak. This review explores diagnostic assays of use in EVD in both resource-rich and resource-limited settings, including their respective limitations, and some novel assays and approaches that may be of use in future outbreaks.

## Introduction

The 2013–2016 Ebola virus (EBOV) outbreak centred in West Africa is the largest ever recorded and has resulted in a substantial global response, involving 77 centres across Guinea, Sierra Leone, and Liberia. The outbreak, which exceeds 28,500 cases [1], has seen sporadic cases imported to resource-rich settings, such as the United Kingdom, the United States, Spain, Switzerland, and Italy, either in the form of repatriation of confirmed cases or by case identification after travellers fell ill after returning from West Africa. Secondary transmission has been recorded in the US [2] and Spain [3]. Although the World Health Organization (WHO) has declared the outbreak over in West Africa, enhanced surveillance has identified sporadic cases, all of which have been rapidly contained. These ongoing intermittent cases occur mostly from sexual transmission and are likely to continue for some time.

Diagnostic and other assays play a vital role in confirming or excluding suspect cases, monitoring disease progression and complications, and discharge planning. In both resource-rich, and resource-limited settings, this allows the appropriate segregation of confirmed cases and quarantine of contacts as well as optimal care and the appropriate allocation of resources.

Sporadic outbreaks of human EBOV infection have appeared with increasing frequency since the virus was first identified in 1976 [4,5]. Person-to-person transmission occurs via direct contact with blood and body fluids [6], and nosocomial transmission is a prominent feature of outbreaks [7]. Indeed, in the West African outbreak, at least 881 health care workers became infected, of which 513 died [8].

Following an incubation period of 2–21 days, patients initially present with nonspecific symptoms, including fever, headache, malaise, and myalgia. By days 3–5 of the illness, a gastrointestinal stage develops with epigastric pain, hiccups, nausea, vomiting, and diarrhoea [9]. Large volume, watery diarrhoea of five litres or more daily has been reported [10]. By days 7–10, neurological manifestations, including delirium, confusion, slowed cognition, or agitation and seizures, may present [10]. Purpuric rash, conjunctival injection, and oozing from venous catheter sites may occur. Massive haemorrhage from the gastrointestinal tract is rare and normally only occurs in fatal cases [11]. For this reason, the term Ebola virus disease (EVD) is now more widely used than the previous term Ebola haemorrhagic fever. Case fatality rates (CFR) of previous outbreaks have been as high as 80%–90%, though most have been between 35%–75% [12]. The CFR in this outbreak is estimated to be below 50% by WHO [1].

Several systemic manifestations are seen in EVD. These may be due to a combination of dehydration, endothelial damage, disseminated intravascular coagulation (DIC), septic shock, and organ damage caused by direct viral infection and associated immune responses [13]. The ability to monitor relevant laboratory parameters is essential for optimal care and can indicate individual patient prognosis. Diarrhoea and volume depletion, likely combined with direct viral renal damage, lead to electrolyte derangement, which may be severe and life-threatening [10,13,14]. Hepatocellular damage is a common feature, and deranged liver enzymes are well documented in animal models and human cases [15,16]. Raised serum levels of creatine kinase (CK), which may be measured within a liver function test panel, are also noted to be a marker of severe disease in viral haemorrhagic fevers (VHF), such as Crimean Congo Haemorrhagic Fever (CCHF) [17]. Disruption to normal coagulation processes is common [16,18]. Biomarkers of deranged coagulation, such as prolonged prothrombin and activated partial thromboplastin times (PT and APTT, respectively), and DIC, such as the presence of fibrinogen degradation products, are noted [15,16]. Aside from the enumeration of the number of platelets, a full blood count (FBC) may show both leukopenia [16] and neutrophilia, the latter in the presence of a subsequent bacterial sepsis or advanced disease. Anaemia and abnormal erythrocyte indices will be observed with blood loss. An inability to monitor and correct the complications of EVD, in particular electrolyte imbalance, may have contributed to the high mortality rates early in the West Africa outbreak.

Access to rapid, accurate diagnostic assays is essential to enable appropriate patient management and may be used to allow effective discharge planning. In addition, effective outbreak control requires the rapid diagnosis, isolation, and treatment of infected individuals, and the follow-up of their contacts. Early containment of the West Africa outbreak was undoubtedly hampered by a lack of rapid diagnostics that could differentiate EVD from other diseases, given its nonspecific clinical features. In both resource-rich and resource-limited settings, the predominant issue at the outbreak onset was limited or absent access to diagnostics allowing EVD to be differentiated from other febrile illnesses. Whilst patients in resource-rich settings remained in isolation facilities for significant periods of time, often blocking side rooms in busy emergency departments, the impact in resource-limited settings was significantly more severe where case identification and subsequent outbreak response became even more challenging. Patient management in Ebola Treatment Centres (ETC) in resource-limited settings is challenging for a number of reasons: large numbers of patients, few staff, and oppressively hot and cumbersome personal protective equipment that limits the time that clinical and support staff can spend in the clinical areas. Additionally, note-keeping is difficult due to infection control limitations, and the use of needles and other sharps pose a significant risk to health care workers. Here, we review the assays currently available in resource-rich and resource-limited settings as well as some in development that could play a role in the diagnosis and clinical management of EVD.

### Diagnostic Methods in a Resource-rich setting

#### Laboratory Requirements

**Fig 1:**
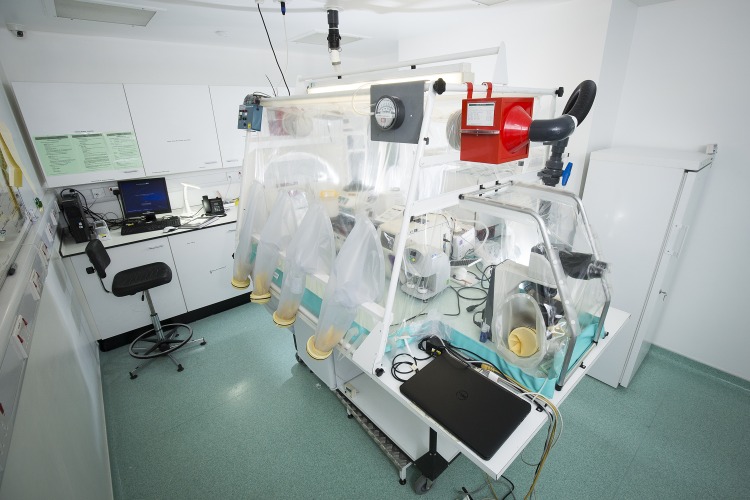
The laboratory isolator at the HLIU of the Royal Free Hospital, London. Image Credit: David C Bishop.

EBOV is categorised as a high-hazard pathogen that is handled at Biosafety Level 4 in the US [19] and is designated as a Hazard Group 4 Pathogen in the UK [20]. Complex facilities are required to examine samples that contain this and similar viruses. These laboratory suites consist of large, highly secure facilities with restricted access, carefully controlled air-handling systems with filtered air, and the meticulous control and inactivation of hazardous waste. Staff are highly skilled and often train for years to be able to work at this level of containment. The manipulation of these samples within these laboratories additionally requires either the isolation of the scientist via suited systems or of the specimens themselves with the use of microbiological safety cabinets. Building, commissioning, maintaining, and staffing these units is expensive and labour intensive, so work on such high-hazard pathogens is usually restricted to a few national or regional centres in resource-rich settings. In the UK, bespoke Containment Level 3+ pathogen laboratories are also located within the High Level Isolation Units (HLIU), Royal Free Hospital (London) (Fig 1), and the Royal Victoria Infirmary (Newcastle), the national centres for managing confirmed cases of VHF. Whilst these facilities do not provide Containment Level 4 viral diagnostics per se, they enable staff to provide pathology assays to exclude alternative diagnoses, such as rapid malaria diagnostics and blood cultures, and to support patient clinical management decisions. These facilities are staffed by trained scientific staff and are available at all times.

### Ebola-specific diagnostics and exclusion of alternative diagnoses

A febrile patient returning from an area endemic for EVD within 21 days of exposure requires rapid, accurate, and safe diagnostic tests not only for VHF but also for more common diagnoses, such as malaria, typhoid, and arboviruses, such as dengue. Samples are taken by medical staff wearing appropriate personal protective equipment and sent to a central facility in appropriate packaging (UN2814 [category A] or UN 3373 [category B] depending on the risk assessment) and using a preapproved courier service [21].

Historically, a combination of antigen and antibody detection via enzyme-linked immunoassay (EIA) and reverse transcriptase polymerase chain reaction (RT-PCR) was used to identify an outbreak in May 1995 in Zaire [22,23]. Electron microscopy (EM) together with antigen and antibody analysis was used to identify a new variant of Ebola in the Ivory Coast in 1995 [24]. The use of RT-PCR in the detection of EBOV has been further described in these outbreaks, as well as for the detection of Ebola Reston [25], and is now recommended [26]. Molecular assays have advantages over the previous technologies of EIA, EM, and immunofluorescence (IF), including increased sensitivity, specificity, and faster turnaround, allowing clinical teams to act upon the results much more rapidly.

The preferred diagnostic method is via direct detection of viral RNA using a nucleic acid amplification test (NAAT) (Table 1). Viral nucleic acid is extracted inside the containment laboratory prior to amplification and detection. An additional advantage of this setup is that additional differential diagnoses can be investigated at the same time. Travellers returning to the UK from VHF-endemic areas will be tested for a panel of likely pathogens (Table 2). The Public Health England Rare & Imported Pathogens Laboratory (RIPL) performs a range of these molecular assays according to patient epidemiology, including pan-Ebola–[27] and Ebola Zaire–specific [28] NAATs. Assays for other geographically relevant diseases that may present in a similar fashion (such as malaria, Lassa fever, or dengue) are performed in parallel. Result turnaround times are minimised to permit the de-escalation of patient isolation and specimen containment or, if positive, expedite transfer to the HLIU at the Royal Free or Royal Victoria Hospitals.

**Table 1:** Summary of diagnostic NAATs.

Company	Assay name	Assay description and target*	Limit of detection	Approx. time to result	Practical considerations	Stage of development/reference
Altona (Hamburg, Germany)	• Real Star Filovirus Screen • Real Star Ebolavirus	• Real-time NAAT targeting the L gene of all five EBOVs • Real-time NAAT for detection and differentiation of all five EBOVs	• 3.16 copies/μL† • 116–675 copies/μL† • 1,250 copies/mL [29]	3–4 hours	-20°C storage and a moderate level of laboratory training required	•CE-IVD marked •Emergency Use Authorization from the US Food and Drug Administration (FDA)
BioMerieux (France)	BioFire Film Array Biothreat-E test	Real-time NAAT for detection of ZEBOV blood and urine within approximately one hour	• 600,000PFU/mL† •400 TCID_50_/mL [30]	2 hours	Ambient temperature reagents and a moderate level of laboratory training required	Emergency Use Authorization from FDA [31]
Cepheid (US)	Xpert Ebola Assay	Real-time NAAT for detection of ZEBOV blood and urine	232 copies/mL† [32]	100 minutes	Ambient temperature reagents; low level of laboratory training required	Emergency Use Authorization from FDA
Trombley assay	Various	Noncommercial pan-VHF multiplex NAAT	0.001–1.0 PFU/PCR [28]	3–4 hours	-20°C storage and a moderate level of laboratory training required	[28]
Panning Assay	Various	Noncommercial pan-Filovirus multiplex NAAT targeting L gene	10 copies per assay [27]	3–4 hours	-20°C storage and a moderate level of laboratory training required	[27]
Roche (Switzerland) and TIB MOLBIOL GmbH (Germany)	LightMix Ebola Zaire rRt-PCR	• Real-time PCR targeting the L gene • Up to 96 results in just over three hours and is compatible with their LightCycler 480 or Cobas z 480 instruments	• 4,781 PFU/mL† • 1,250 copies/mL [29]	>3 hours	Ambient temperature reagents (≤24°); moderate level of laboratory training required	Emergency Use Authorization from FDA; CE marked, but has not yet been cleared or approved for general use by the FDA
Biocartis, Janssen Diagnostics and the Institute for Tropical Medicine in Antwerp (Belgium)	Idylla system	Real-time NAAT on a fully automated molecular diagnostic platform using 0.2 mL of blood	465 PFU/mL†	100 minutes	Ambient temperature reagents (≤30°); moderate level of laboratory training required	CE-IVD marked in Europe

*where provided in manufacturer† manufacturer’s data

PFU = plaque forming unit, TCID_50_/mL = 50% tissue culture infections dose, PCR = polymerase chain reaction, ZEBOV = Zaire EBOV.

**Table 2:** Differential diagnosis of febrile illness on return from West Africa.

Differential diagnosis of febrile illness on return from West Africa	Method of detection
Typhoid	Culture
Malaria	Microscopy, rapid chromogenic test, NAAT
Lassa Fever	NAAT
Dengue	NAAT
Chikungunya	NAAT
Ricketsia	NAAT
Rift Valley Fever	NAAT
Crimean Congo Haemorrhagic Fever	NAAT

As patients may have an undetectable viraemia in the early stages of disease, it is recommended that tests should be repeated if the clinical suspicion of EVD remains until 72 hours into the illness [26]. However, this approach may alter when more data becomes available.

### Disease management

#### NAAT interpretation and semi-quantitative viral loads

Many NAATs, such as those listed in Table 1, provide not only a qualitative positive/negative result but also an indication of the number of virions within the sample. The presenting viral load has prognostic value, for which high viral loads are associated with higher mortality [14,33,34], whilst serial testing is useful to chart clinical progress and to inform decision-making around novel therapeutic interventions. Slight variation in assay quantitative results may arise depending on sample type (assays are usually optimised for EDTA plasma rather than serum), but, regardless of sample type, serial assays over the course of illness provide very valuable prognostic information. Whilst discharge planning is often considered when sequential daily blood samples performed in parallel fail to amplify above the lower limit of detection (LLOD), result interpretation can prove difficult because a low-level viraemia can persist for many days (and the LLOD may vary between assays). As the patient improves clinically, NAAT testing of other anatomical sites may inform the infection risk assessment prior to patient de-isolation, although more data are needed to understand the relationship between positive tests for EBOV RNA in these sites and risk of transmission. These samples include sputum, throat swabs, sweat, urine, and breast milk, whilst the more intimate samples of semen and cervical samples can be deferred to the outpatient clinic setting.

### Supportive pathology assays

Measuring biomarkers of the complications of EVD is imperative to monitoring disease progression and assists clinical teams to correct such abnormalities. A nonexhaustive panel of suggested parameters is listed in Table 3. The ability to analyse these parameters in resource-rich settings is well established, either in centres of excellence that manage confirmed cases of VHF infection or in routine diagnostic laboratories. Whilst diagnostic assays are awaited, the authors feel that supportive pathology assays may be analysed safely using routine processes and autoanalysers following appropriate risk assessment [21]. This would enable optimal patient care whilst EVD diagnostic assays are performed. When reviewing previous imported cases of VHF to resource-rich settings, supportive assays have been performed using routine analysers in standard pathology laboratories, often prior to the diagnosis being made with no recorded transmissions to laboratory workers [35–39]. In addition, over 9,000 cases of CCHF were notified in the whole of Turkey (2002–2014), with an estimated minimum of 180,000 blood samples processed in routine laboratories with no additional precautions. A review was performed of 51 health care exposures that occurred in nine centres where 4,869 of these patients were managed. Only two cases in laboratory staff were identified. One may have been associated with phlebotomy and the other with handling samples whilst not wearing protective gloves [40].

**Table 3:** Summary of suggested assays for the management of EVD patients.

Biochemistry	Haematology	Coagulation
Sodium	Haemoglobin	Prothrombin Time (PT)
Potassium	Platelets	Activated Partial Thromboplastin Time (APTT)
Urea	Leucocyte count with differential	Fibrinogen degradation products
Creatinine	Erythrocyte count	Thromboelastography (TEG)
Aspartate transaminase (AST)		
Alanine aminotransferase (ALT)	Blood grouping & antibody screening	
Bilirubin	Cross matching	
Alkaline phosphatase		**Others**
C-reactive protein		Malaria
Lactate		HIV
Blood gases		Microscopy, culture, and sensitivity, especially of stool and blood cultures
Magnesium		Pregnancy test

**Fig 2:**
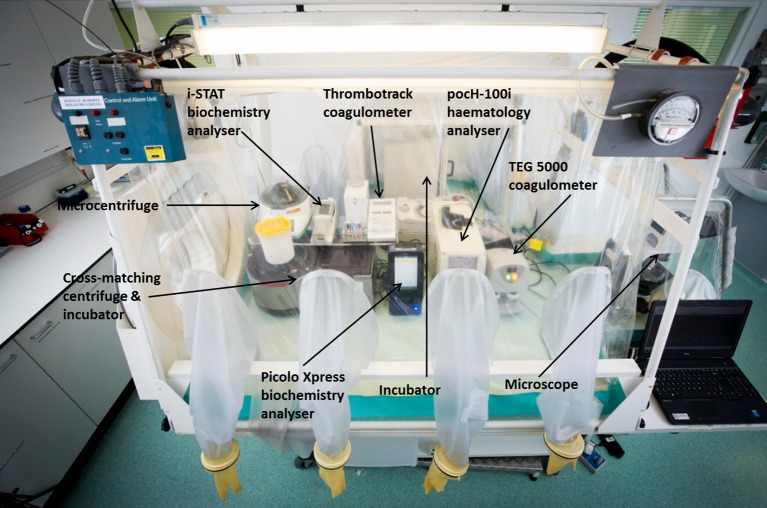
Layout of POC analysers within the laboratory isolator at the HLIU of the Royal Free Hospital, London. Image Credit: David C Bishop.

Stand-alone, discrete analysers may be used in high-level containment units either in combination with suited systems, safety cabinets, or isolators to protect staff. The HLIU at the Royal Free uses small, Point of Care (POC) analysers in a designated laboratory to provide the assays listed in Table 3, and some suitable analysers that meet these needs are shown in Table 4 and Fig 2. It is noteworthy that the performance of POC analysers may vary significantly from routine autoanalysers. The benefits of decreased size, portability, and ease of use should be considered alongside the potentially poorer accuracy and precision of these assays. It is also important to remember that assay methodologies, and, therefore, normal ranges, vary between instruments. Therefore, results obtained on an autoanalyser in a routine laboratory may not be directly comparable to the results obtained on POC instruments in a specialised centre.

**Table 4:** Some examples of POC analysers suitable for measuring the analytes listed in Table 3.

Haematology analysers	Methodology	Biochemistry analysers	Methodology	Coagulation analysers	Methodology
pocH-100i (Sysmex, UK)	Quantitative, automated cell counter with leucocyte differential measured by electrical impedance	Piccolo Xpress (Abaxis, US)	Colourimetric end-point and rate reactions using lyophilised reagents in single-use discs in a small, bench-top analyser	i-Stat (Abbott Diagnostics, US)	Handheld ion-selective electrode analyser with single-use cartridges
Horiba ABX systems (Horiba, UK)	Quantitative, automated cell counter with leucocyte differential measured by electrical impedance	i-Stat (Abbott Diagnostics, US)	Handheld ion-selective electrode analyser with single-use cartridges	Thromotrack (Abaxis, UK)	Measurement of clot formation using a ball-bearing and magnet. Measures PT/INR and APTT
Beckman Coulter Ac.T systems (Beckman Coulter, UK)	Quantitative, automated haematology analyser and leucocyte differential counter			TEG 5000 (Haemonetics Corporation, Braintree, Massachusetts)	Thromboelastography: Measurement of clot formation around a thin wire probe, assessing maximum rate of thrombin generation, time to maximum rate of thrombin generation, maximum amplitude of clot formation, and reaction time

### Innovation

Due to the size of the West Africa outbreak, and, therefore, the large number of individuals entering the UK who were assessed to be at risk of EVD, there was a need to increase testing capacity and reduce turnaround times. Public Health England introduced diagnostic testing to a number of regional centres with the use of a commercial assay (BiofireFilmArray, BioFire, Utah, US) (Table 1). This real-time, nested NAAT allows amplification and detection of Ebola Zaire in approximately two hours. Whilst turnaround times fell for Zaire EBOV, mainly due to reduced transport times to the testing laboratories, samples still needed to be referred to RIPL, the central UK facility, if a full, geographically restricted panel was required. However, rapid EVD testing in these regional centres allowed rapid step-down of isolation precautions in a significant number of cases in which EVD was assessed by the UK Imported Fever Service to be the principal differential diagnosis.

### Resource-limited setting

Limited health care and laboratory facilities existed prior to the start of the outbreak. Nightingale-style wards were mostly used, with a paucity of medical and nursing staff. Estimates for Sierra Leone suggest the country is lacking 2,551 doctors and 9,593 nurses to meet WHO minimum recommended staffing levels [41]. Patients were frequently cared for by relatives, and minimal laboratory support was available. The nature of the nonspecific symptoms meant that identifying infected patients early in the outbreak was difficult, with clinical algorithms having low specificity [42]. In previous outbreaks, the limited availability of EVD (and other microbiological and virological) diagnostic assays and facilities resulted in samples being referred to established, overseas research centres, often in retrospect [43–46], with subsequent minimal, positive, real-time impact. This outbreak was no different, with initial samples being analysed in France and Germany [47]. Whilst diagnostic tests were awaited, patients with suspected EVD were often housed in facilities with limited separation between patients, where the risk of nosocomial acquisition of EVD was significant, though evidence suggests such transmission was limited [48,49]. Additionally, patient care (nursing, monitoring, and treatment) is significantly compromised through the very limited time that staff could operate in the clinical areas. There was considerable difficulty, particularly early in the outbreak, in establishing safe and robust specimen transport mechanisms to EVD-testing laboratories. Turnaround times for EVD diagnostic results were very slow, often several days at the beginning of the outbreak, until several EVD testing laboratories were set up. Many organisations that ran ETC did not have access to supportive diagnostics. This meant that patients received syndromic, standard care, which was not individualised to need.

### Laboratory requirements

**Fig 3:**
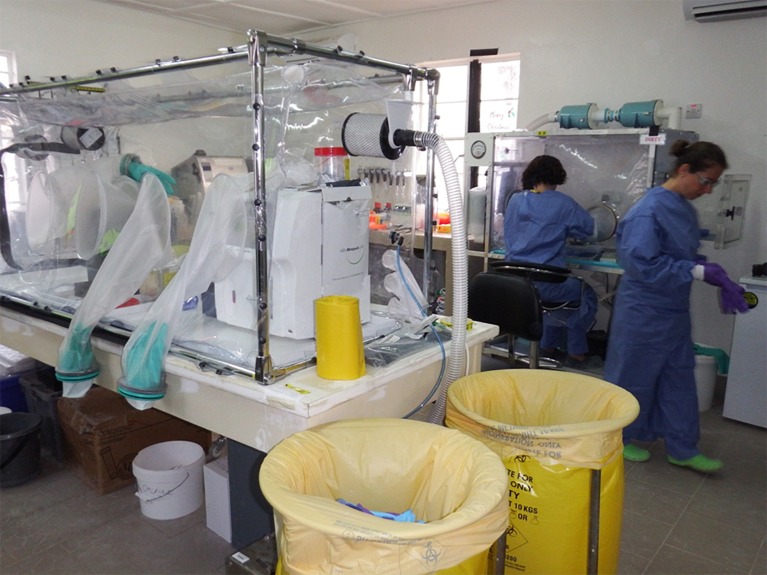
Public Health England Laboratory at the Kerry Town ETC, Sierra Leone. The POC analysers are situated in the isolator in the foreground. The isolator in the background is used for virus inactivation and RNA extraction. Image supplied by A. J. Simpson.

Following the onset of the Ebola outbreak, at least nine ETC were set up in Sierra Leone by nongovernmental organisations (NGOs), such as Médecins Sans Frontières, Save The Children, International Medical Corps, and Goal, and the British and Sierra Leone Armed Forces, with similar construction happening in Liberia and Guinea. Much like a resource-rich VHF containment facility, these were arranged as a one-way flow to separate uncontaminated health care workers (entrance, eating and changing facilities, pharmacy) with intermediate zones (containing auxiliary services such as wash teams and clinical handover stations), suspect patient Ebola Holding Units (EHUs, which were also stand-alone facilities attached to government health facilities), and red zones (confirmed infected patient wards). Health care workers returned back to the green zone via a doffing and decontamination area and showers. Laboratory facilities were often located in intermediate zones so they could safely receive samples from both the red zone of the ETC and the community. Such facilities were in newly erected, repurposed hospitals or temporary buildings that do not conform to the strict containment laboratory standards that would be required in a resource-rich setting (Fig 3). For example, these facilities would generally not be under negative air pressure or be sealable for fumigation. Some may not have unidirectional staff flow with separate entrance and exits with showers.

The challenges of providing diagnostics in such settings are numerous, including power cuts that interrupt both diagnostic assays and cold storage of molecular reagents and high environmental temperatures that may interfere with the optimal performance of some NAAT platforms and other POC analysers. The provision of power generators and air conditioning was necessary to ensure continuity of laboratory services. Like the clinical facilities, laboratories produce considerable volumes of hazardous waste, which must be stored safely until appropriate inactivation and disposal can be arranged.

### Ebola-specific diagnostics and exclusion of alternative diagnoses

As a result of the international response, molecular methods for the detection of Ebola are now widely used and have been utilised in field hospitals set up in response to previous outbreaks (Table 1). Assays in use in West Africa include the Trombley assay [28] and the RealStar Filovirus Screen RT-PCR (Altona, Hamburg, Germany). Such technologies are not always easy to use in relatively unskilled hands, requiring infrastructure, training, and often the assistance, at least initially, of international staff and resources.

Following the international response, NAATs were deployed in laboratories as they were established by governmental agencies and nongovernmental organisations. Rapid turnaround times were desirable to enable the isolation of infected patients and the discharge of negative patients from high-risk exposure settings of the Ebola holding centres. Comparison of sequential cycle threshold (Ct) values was difficult, as different platforms were often used between the referring unit and receiving ETC. Unlike resource-rich settings, sequential Ct values were performed less frequently. Repeat NAATs were usually performed in ETC to assess patient recovery and to assess whether a patient could be discharged. As in resource-rich settings, an undetectable blood viral load together with clinical resolution of disease was used as an indication for patient discharge in Monrovia, Liberia [10], and in Sierra Leone. Facilities to investigate alternative diagnoses were largely absent. Other diagnostics were usually limited to malaria rapid diagnostic tests (RDT) [50], meaning that other potentially serious diagnoses such as typhoid, dengue, and Lassa fever could well go unrecognised. EVD assays can also be used to assess the efficacy of environmental decontamination procedures within EHUs and ETC [51].

### Supportive pathology assays

The provision of supportive assays in a resource-limited setting is challenging and is unavailable in most settings due to expensive equipment and reagents, availability of trained staff to perform the assays and interpret the results, provision of continuous power supply, and refrigeration and appropriate environmental temperatures. The Piccolo (Abaxis, US) (Table 4) or Fuji Dri-Chem NX-500 were used to measure biochemistry parameters in this outbreak in Kenema, Sierra Leone [14] as well as in Kerry Town, where it was used alongside the Horiba ABX systems (Horiba, UK) for the measurement of FBC. The i-Stat (Abbott Diagnostics, US) was used in Conakry, Guinea to measure basic biochemistry, coagulation, and blood gases [52]. However, this required the establishment of dedicated laboratories and containment facilities, including bespoke sealed, negative pressure, high efficiency particulate air (HEPA) filtered isolators, and trained staff from overseas (Fig 3).

### Innovation

The unprecedented scale and duration of the West Africa outbreak has resulted in the development of new technologies being offered for trial and approval [53] (Tables 1 and 5). Such VHF diagnostic POC tests are simple to use and ideal for resource-limited settings where outbreaks and infections are both sporadic and transient [54]. Handheld lateral flow assays that detect viral antigens in blood and body fluids require no electricity, can often be stored at ambient temperatures, and can provide a result within approximately 10 to 20 minutes. Several are being trialled and marketed currently, examples of which are detailed in (Table 5). Given their reported 100% sensitivity, one significant application is their use as a screening “rule-out” test. This attribute is of considerable importance given the likely need for repeated testing for suspect patients in the region [55]. Additionally, there is a need to perform these assays on samples other than venous blood. When confirming EVD as the cause of death in cadavers, it is safer for health care and public health staff to take oral swabs rather than to obtain blood samples and risk a sharps-related injury. This could also apply to living patients for increased ease and safety, and it has been successful [56,57]. Achieving suitable regulatory approvals for new POC devices has, however, proved to be an obstacle to the rapid deployment of such assays.

**Table 5:** Summary of diagnostic antigen-based assays.

Company	Assay name	Assay description	Stage of development	Performance*	Reference
Corgenix (US)	ReEBOV Antigen Rapid Test	Lateral flow device for the detection of VP40 antigen in blood in approximately 20 minutes	FDA approved. WHO for use in the detection of EVD where it is not possible to use a molecular assay	Sens 100%Spec 92.2%(tested against Altona NAAT)	Field validation [54]; the manufacturer has used the same technology for the detection of Lassa fever virus [58]
Chun-Yan Yen and colleagues	Multiplexed lateral flow assay	Silver nanoparticles conjugated to detect EBOV GP (as well as Dengue and yellow fever viruses)	Undergoing field studies		[59]
Stada Pharm and Senova (Germany)	Ebola lateral flow test	Handheld lateral flow device for the detection of EBOV antigens in blood and body fluids in 10 minutes	Commercially available		
France's Atomic Energy Commission (CEA) with Vedalab	Ebola eZYSCREEN lateral flow assay	Handheld lateral flow device for the detection of EBOV antigens in blood, plasma, and urine in less than 15 minutes	Commercially available		
The United Kingdom’s Defence Science and Technology Laboratory (DSTL)	Lateral Flow assay	Semi-quantitative detection of undisclosed EBOV antigen using capillary blood in 20 minutes		Sens 100%Spec 96.6%	[55]

*if available in published literature or from the manufacturer.

VP40 = viral protein 40, GP = glycoprotein.

Rapid molecular detection of EBOV has been demonstrated using Reverse Transcription-Loop-Mediated Isothermal Amplification (RT-LAMP) assay [56]. This assay allows amplification and detection in approximately 35 minutes with excellent sensitivity and specificity, although viral nucleic acid extraction must initially be performed. Other novel technologies, such as portable and low-cost molecular systems [60], handheld NAAT devices [61], and the use of nanoparticles to detect isothermally amplified nucleic acids [62], have been described. Another innovative molecular assay showing promise is the feasibility of providing real-time genome sequencing in an outbreak setting [63]. As equipment becomes more portable and data analysis becomes faster, it is likely that real-time genome sequencing of future outbreaks will bring real benefit in terms of epidemiology and monitoring viral variation.

## Discussion

The unparalleled nature of this outbreak, in terms of case numbers, geographic location, and size, has generated many challenges in both resource-rich and resource-limited settings. All health care systems have a need to rapidly identify and isolate patients, provide the best possible care, prevent onward transmission (including to health care workers), and provide safe discharge planning. High-quality pathology assays underpin all of this.

Resource-rich health care facilities experienced an unprecedented demand for assessment and investigation of unwell individuals returning from West Africa. Access to safe diagnostics and supportive assays in a general hospital setting must be further expanded. Facilitating rapid turnaround and (usually) patient de-escalation should be a priority to limit the use of limited resources such as emergency side rooms.

Health care systems in resource-limited settings inevitably struggled to cope even when augmented with overseas aid. Impetus must now focus on how to develop the laboratory infrastructure: what will happen to the new laboratories that have been established (often in temporary buildings), and how will the expertise be maintained and transferred to local staff? Public Health England is committed to continuing to provide EVD diagnostics in Sierra Leone for the foreseeable future. As part of the ongoing surveillance efforts, and with other UK partners, including the Department for International Development (DFID), it intends to build, equip, and operate new, modern laboratory facilities in regional hospitals (including training and mentoring of local staff).

There is undoubtedly a role for novel diagnostics in these settings to tackle future outbreaks of this and other diseases. There is a need to roll out assays as rapidly as possible following their efficacy being established and accepted. This has been argued to be too slow in this outbreak with regards to lateral flow assays [64]. The increased availability of reliable, user-friendly assays in future outbreaks could have a great benefit in limiting the number of isolation beds required for EVD testing, controlling transmission, and improving patient outcomes. Moreover, traditional microbiological and virological assays need to be deployed across the region to allow for diagnosis of a variety of parasitic, bacterial, and viral pathogens. This will allow for alternative diagnoses of unexplained febrile illness to be rapidly made as well as provide a baseline for disease prevalence in the affected countries.

There is a continued need for investment in this area—new assays need to be developed, evaluated, and embedded into local health care systems to allow the prompt control of future outbreaks. For instance, rapid, low-cost molecular detection of Zika virus has been achieved using programmable, biosynthetic components, which may be applicable in an outbreak setting [65]. Additional vigilance and surveillance is required to ensure that such assays meet the diagnostic needs as new viruses such as Bundibugyo EBOV are discovered [66] and inevitable sequence variation occurs [67–70]. Finally, up-skilling and retention of laboratory staff, along with sustained resourcing of basic pathology services, needs to be at the fore of long-term resilience plans. The recent Ebola outbreak resulted in numerous lessons learned and significant innovation. As a result, it is hoped that future outbreaks will be identified faster and ultimately terminated more efficiently in part through greater access to portable, easy-to-use diagnostic assays.

**Key Learning Points**

- The initial identification and containment of this outbreak was hampered by poor access to diagnostic assays.
- Patients with Ebola in West Africa were often managed in the absence of supportive pathology assays, which may have led to suboptimal care.
- There is a need to roll out diagnostic and supportive assays much more quickly in the next such outbreak, including the rapid establishment of clinical trials for new technologies.
- Unwell travellers who returned to resource-rich settings from West Africa were often isolated for some time (and investigations/treatment delayed) whilst Ebola assays were performed in reference facilities.
- Unwell travellers who are assessed to be at high risk for Ebola and other VHF can be managed safely in resource-rich settings whilst diagnostic results are awaited. This includes the processing of routine pathology assays.

**Top Five Papers**

1. Baize S, Pannetier D, Oestereich L, et al. Emergence of Zaire Ebola virus disease in Guinea. N Engl J Med. 2014 Oct 9;371(15):1418–25. Epub 2014 Apr 16.
2. Kortepeter MG, Bausch DG, Bray M. Basic clinical and laboratory features of filoviral hemorrhagic fever. J Infect Dis. 2011;204 Suppl(Suppl 3):S810–6.
3. Fletcher TE, Fowler R a., Beeching NJ. Understanding organ dysfunction in Ebola virus disease. Intensive Care Med. 2014;40(12):1936–9.
4. Schieffelin JS, Shaffer JG, Goba A, Gbakie M, Gire SK, Colubri A, et al. Clinical Illness and Outcomes in Patients with Ebola in Sierra Leone. N Engl J Med. 2014;371:2092–100.
5. Racsa LD, Kraft CS, Olinger GG, Hensley LE. Viral Hemorrhagic Fever Diagnostics. Clin Infect Dis. 2016 Jan 15;62(2):214–9.
